# Genome Sequences of Mycobacteriophages Jane and Sneeze, New Members of Cluster G

**DOI:** 10.1128/genomeA.01486-16

**Published:** 2017-03-16

**Authors:** Catherine M. Mageeney, Cimrin Bhalla, Charles A. Bowman, Bhavishya Devireddy, Adrienne P. Dzurick, Lee H. Graham, Marina Grossi, Margaret A. Kenna, Mikala A. Kowal, Drew D. Nielsen, Rachel A. Pallay, Kaitlyn M. Ruffing, Daniel A. Russell, Samantha L. Sarli, Adama Shaw, Joseph W. Skibbens, Joseph N. Teyim, Vassie C. Ware

**Affiliations:** a Department of Biological Sciences, Lehigh University, Bethlehem, Pennsylvania, USA; b Department of Biological Sciences, University of Pittsburgh, Pittsburgh, Pennsylvania, USA

## Abstract

Jane and Sneeze are newly isolated phages of *Mycobacterium smegmatis* mc^2^155 from Hillsborough, NJ, and Palo Verde, Costa Rica, respectively. Both are cluster G, subcluster G1 mycobacteriophages. Notable nucleotide differences exist between genomes in the right half, including the presence of mycobacteriophage mobile element 1 (MPME1) in Jane.

## GENOME ANNOUNCEMENT

A large number of mycobacteriophage genomes have been functionally annotated (1), allowing for insights into genomic structure, diversity, and genome evolution in this phage group. Further investigation of mycobacteriophage genomes and gene functions may aid in developing new molecular tools for diagnostics and therapeutics for tuberculosis (2, 3).

Mycobacteriophages Jane and Sneeze were isolated by enrichment from soil from Hillsborough, NJ, and Palo Verde, Costa Rica, respectively. Following phage purification and amplification, transmission electron microscopy revealed *Siphoviridae* morphologies. Double-stranded DNA (dsDNA) from each phage was isolated and sequenced by Ion Torrent, and reads were assembled into complete genomes using Newbler and Consed, with 3,162-fold and 2,606-fold coverage for Sneeze and Jane, respectively. The Sneeze genome is 42,429 bp, with 66.5% G+C content and 11-base 3′ overhangs with the sequence 5′-TCCCATGGCAT. Jane has a 41,901-bp genome, 66.6% G+C content, and an 11-base 3′ overhang of 5′-CCCCATGGCAT. The nucleotide difference at the 5′ end of the Sneeze 3′ overhang is not observed in other closely related mycobacteriophages.

Coding DNA sequence (CDS) predictions for both mycobacteriophages were determined using DNA Master (http://cobamide2.bio.pitt.edu/) embedded with GeneMark and Glimmer gene calling algorithms. Sneeze contains 64 predicted protein-coding genes, while Jane contains 63 predicted protein-coding genes. No tRNA genes or transfer-messenger (tmRNA) genes were predicted when analyzed with Aragorn and tRNAscan (4, 5). Functions were attributed to 31 of 64 Sneeze CDSs and 33 of 63 Jane CDSs, using BLASTP and HHPred (6). Sneeze and Jane are cluster G, subcluster G1 mycobacteriophages, bringing the total number of G1 subcluster members to 29 (1, 7, 8) (http://www.phagesdb.org). Jane is 99% identical to BPs (a G1 mycobacteriophage), spanning 99% of the genome. Sneeze is less identical (96%) to BPs, spanning 13% of the genome. Sneeze is most closely related to Taheera, with 97% identity spanning 31.7% of the genome. All but two genes (*gp32* and *gp33*) in Jane and Sneeze are rightward-transcribed.

The first halves of the Jane and Sneeze genomes include CDSs predicted to encode proteins that function in structure and assembly, host integration, and lysis. These gene functions are consistent with those from other G1 subcluster phages and display syntenic relationships across all mycobacteriophage genomes. Many genes in the second half of the genome, as in other G1 phages, cannot currently be assigned a function. Putative functions in the right end of the genome include recombination factors and endonucleases. Additionally, Jane, but not Sneeze, contains a mycobacteriophage mobile element 1 (MPME1) as *gp59*. MPME1 elements, found in many G cluster genomes, are described as “short-terminal inverted repeats” characteristic of other bacterial mobile elements (7). The MPME1 found in Jane is in phamily number 3572, which contains 100 members from various clusters. There are 12 additional G1 subcluster phages with the same MPME1 in the same location as in Jane, including BPs and Hope. Sneeze is one of five G1 subcluster phages that lacks MPME1.

### Accession number(s).

The Jane and Sneeze genome sequences are available in GenBank under accession numbers KX588251 and KX534004, respectively.
